# Greater Mouse‐Tailed Bats Glide During Approximately 50% of Their Flight

**DOI:** 10.1111/nyas.70416

**Published:** 2026-10-13

**Authors:** Mor Taub, Aya Goldshtein, Yotam Orchan, Sivan Toledo, Ran Nathan, Yossi Yovel

**Affiliations:** ^1^ School of Zoology, Faculty of Life Sciences Tel Aviv University Tel Aviv Israel; ^2^ Department of Ecology, Evolution and Environmental Biology Columbia University New York New York USA; ^3^ Department of Ecology, Evolution and Behavior The Hebrew University of Jerusalem Jerusalem Israel; ^4^ Minerva Center for Movement Ecology The Hebrew University of Jerusalem Jerusalem Israel; ^5^ Blavatnik School of Computer Science Tel‐Aviv University Tel Aviv Israel; ^6^ Sagol School of Neuroscience Tel Aviv University Tel Aviv Israel

**Keywords:** bats, echolocation, flapping, flight, gliding, on‐board

## Abstract

Flapping flight is widely regarded as a mechanically complex and energetically demanding mode of locomotion. In bats, the evolution of flapping flight is hypothesized to have arisen from gliding, an unpowered form of aerial movement. Although intermittent flap‐gliding flight is considered advantageous in many bird species, this flight strategy has rarely been studied in bats. In this study, we used on‐board tracking devices and audio recordings to characterize the flight behavior and corresponding echolocation of greater mouse‐tailed bats (*Rhinopoma microphyllum*) in the wild. Our results reveal that these bats consistently integrate gliding into flapping flight, not only through brief, intermittent flap‐gliding intervals but also via prolonged gliding bouts that can be as long as dozens of seconds. Gliding accounted for approximately 50% of the total flight duration and occurred during both commuting and foraging flights, but was rarely observed directly during prey pursuit. Across all flight modes, bats continuously emitted longer echolocation calls at lower calling rates during long gliding bouts. These findings demonstrate substantial flexibility in bat flight behavior and suggest that gliding could have been retained as a functional and integral component of an adaptive flight strategy despite possible constraints on echolocation.

## 1 Introduction

Powered flight in bats represents one of the most complex locomotor adaptations among mammals and underpins the ecological success and extraordinary diversification of the order Chiroptera [1]. Most studies of bat flight have focused on continuously flapping locomotion [2, 3], emphasizing wing kinematics [4, 5, 6], structure [7], and metabolic costs [8, 9] associated with sustained powered flight. However, theoretical studies indicate that bats are not restricted to strictly continuous flapping [10, 11, 12]. Several species of Pteropodidae incorporate gliding and/or soaring into their flight behavior [10, 13, 14], mostly soaring on thermal air currents when flying during the day. Other anecdotal observations indicate that several insectivorous bats intermittently incorporate short gliding intervals into their flight, resulting in an intermittent flap‐gliding flight pattern [15]. Despite these observations, short gliding flights were only documented in a few species [16, 17, 18].

Gliding occupies a central position in hypotheses concerning the evolutionary origin of bat flight [1, 19, 20, 21, 22, 23, 24, 25, 26]. Most models posit that powered flight evolved from an ancestral gliding stage, with progressive development of aerodynamic control and muscular power [20]. The switch to powered flight likely also played an important role in the evolution of echolocation, which is coupled to the wingbeat in order to save energy [22, 27, 28]. Gliding is a much less demanding, unpowered form of flight [25]. Contrary to continuous flapping, during gliding, minimum energy is expended by the muscles to keep the wings steady [10]. Gliding flight and intermittent flap‐gliding behavior are very common in birds and were shown to reduce energy expenditure compared to continuous flapping [29, 30, 31, 32]. Brief gliding intervals were also theorized to provide energetic or control‐related benefits during the flight of the common pipistrelle (*Pipistrellus pipistrellus*), particularly in cluttered environments or during maneuvering flight [16].

In this study, we examine the flight behavior of the greater mouse‐tailed bat (*Rhinopoma microphyllum*), focusing on the occurrence and characteristics of gliding flight modes under natural conditions. The greater mouse‐tailed bat is adapted for open‐space foraging and is characterized by long, narrow wings with relatively high aspect ratio and wing loading compared to many clutter‐adapted bats, traits associated with fast, efficient flight and reduced maneuverability [15, 33]. They hunt on the wing [15] and exhibit foraging bouts in between commuting flights [34]. Field observations and Global Positioning System (GPS) studies indicate that *R. microphyllum* employs undulating flight trajectories [12, 35], incorporating brief nonflapping phases that resemble intermittent gliding [15]. We used on‐board tracking, measuring the bats’ *z*‐axis acceleration in order to characterize three different flight modes: flapping, short‐duration gliding, and long‐duration gliding that were observed during commuting (straight flight) and foraging (tortuous flight).

Because bats couple their echolocation emission to the wingbeat in order to save energy [27, 28], gliding intervals might interact with echolocation [16]. For that reason, we used an ultrasonic microphone to record the bats’ echolocation calls during flight and further examined the relationship between different flight modes, that is, flapping or gliding, and echolocation characteristics. We hypothesized that the bats would continue to call during all flight modes but alter their echolocation calls in accordance with the specific flight mode, that is, reduce calling rate during gliding, in order to conserve energy.

## 2 Materials and Methods

Experiments were conducted under permits from Tel‐Aviv University IACUC (Number: L‐15‐045) and the Israel Nature and Parks Authority (Numbers: 2018/41843, 2019/42193).

### 2.1 Monitoring Bats’ Movement and Echolocation

The movement of five greater mouse‐tailed bats (*Rhinopoma microphyllum*) was recorded using a high‐resolution, real‐time reverse‐GPS system that localizes the position of the bats using lightweight tags (ATLAS system, Israel [36]). An additional on‐board device was attached to the bats to record their echolocation and acceleration (Vesper, ASD Ltd, Israel). The total mass of the tags (Atlas and Vesper together) was 4 ± 0.1 g (*n* = 5), which accounted for 12 ± 2% of the total body mass of the bats (32.2 ± 6 g). Although the tag mass was relatively high, the tagging duration was short, and previous studies validated that *R. microphyllum* can fly and maneuver properly during foraging while carrying an on‐board tracking device weighing up to 14% of their body mass with minor impact [37] (the same was shown for other species [38, 39]). The bats were captured in June/August 2018 (Table S1), a few hours before sunset, tagged, and released individually during evening emergence, allowing them to forage naturally with the rest of their colony (see detailed description in [40]). The bats were recaptured within 1–4 days, and the device was removed.

### 2.2 Movement Analysis

Bats’ localization was sampled every 1–2 Hz. However, not all samples yielded successful localizations, and the raw data included both low‐ and high‐quality localizations. Thus, we first filtered the tracking data to remove locations with unreliable positional estimates. For each localization, we calculated a confidence score ranging from 0 to 2, using both the number of ATLAS stations contributing to the localization and the estimated standard deviation (SD) of the position estimate (for details on ATLAS accuracy, see [40]). Localization detected by at least seven stations, or with an estimated positional SD below 80, received the highest confidence score of 2. Localizations detected by three to six stations were assigned a score of 1, whereas all other locations were assigned a score of 0. Sequences of at least five consecutive points with a confidence score of 1 were reassigned to a score of 2. We then retained only locations with a confidence score of 2 for subsequent analyses.

Additional filtering was applied to remove unlikely movement estimates. A point was discarded if it met any of the following conditions: localization‐quality SD greater than 40, ground speed greater than 20 m/s, or a displacement of more than 500 m relative to at least one of the five preceding points. This filtering resulted in an effective median sampling interval of 4 s across the five bats. Tracks were subsequently smoothed with a LOESS filter [41] (9‐point window) and linearly interpolated to yield one position every 4 s (Figure S1).

#### 2.2.1 Classification of Commuting and Foraging

Each localization point was classified as either commuting or foraging using the straightness index (SI) [42], which measures the ratio of the direct bee line to the actual flight distance within ∼1‐min (64 s) intervals. We chose this duration for several reasons. First, our aim was to characterize flight behavior at a coarse temporal scale, capturing broad movement patterns between sites rather than the fine‐scale dynamics of individual feeding attempts. This interval‐window was chosen specifically to exclude short‐scale search behavior within a foraging patch, and to instead isolate longer commuting bouts between sites; a finer resolution would risk misclassifying transient within‐patch search movements as commuting. Second, a 1‐min interval between fixes still represents a comparatively high‐resolution sampling rate for characterizing broad‐scale flight behavior. Finally, using shorter intervals would increase the risk of autocorrelation between consecutive fixes, which can arise at high sampling rates and would violate the independence assumptions underlying our analysis. Points with a straightness index of 0.5 or higher were categorized as commuting, while those below were categorized as foraging. This threshold was set based on a nearly bimodal distribution of their straightness index (Figure S2) that strongly suggests two modes of movement—straight and tortuous. This bimodality is consistent with previous work on this species [35], supporting SI as a biologically meaningful criterion for segmenting commuting from foraging flight. Data classified as feeding approaches (attacks—see below) were excluded from both commuting and foraging for all analyses and analyzed separately.

#### 2.2.2 Classification of Flapping, Gliding, and Long‐Gliding Bouts

Bats’ flight mode (flapping vs. gliding) was classified using the vertical acceleration signal from the inertial measurement unit (IMU—zACC), which captures the vertical acceleration generated by the wing‐beat and was measured at 50 Hz. During flapping flight, this signal shows a very clear oscillatory pattern with a frequency of ∼8−12 Hz (Figure 1 and Figures S3 and S4). First, all peaks in the zACC signal were identified using MATLAB's *findpeaks* function with a minimum peak prominence of 100 and a minimum peak distance of 4 samples. Peak prominences were then clustered into two groups using k‐means (Figure S5). The cluster with the higher mean prominence was classified as flapping, whereas the lower‐prominence cluster was classified as gliding. For each zACC peak, behavioral classification (e.g., commuting or foraging) was assigned and synchronized to the movement and audio data streams. The movement‐derived straightness index was linearly interpolated to each zACC peak timestamp, and peaks with a straightness index below 0.5 were classified as foraging. In addition, each zACC peak was matched to the nearest echolocation call, and peaks associated with feeding behavior (the start of the approach, inter‐pulse interval [IPI] ≤ 0.08 s) [40, 43] were classified as attacks (Figure S6). Finally, the peak‐level classifications were grouped into contiguous behavioral segments, with a new segment created whenever the flapping/gliding and/or foraging/commuting label changed. Segments labeled as gliding and lasting more than 10 s were classified as long‐gliding bouts, whereas gliding segments of 10 s or less were classified as short glides (Figure S5B).

**FIGURE 1:**
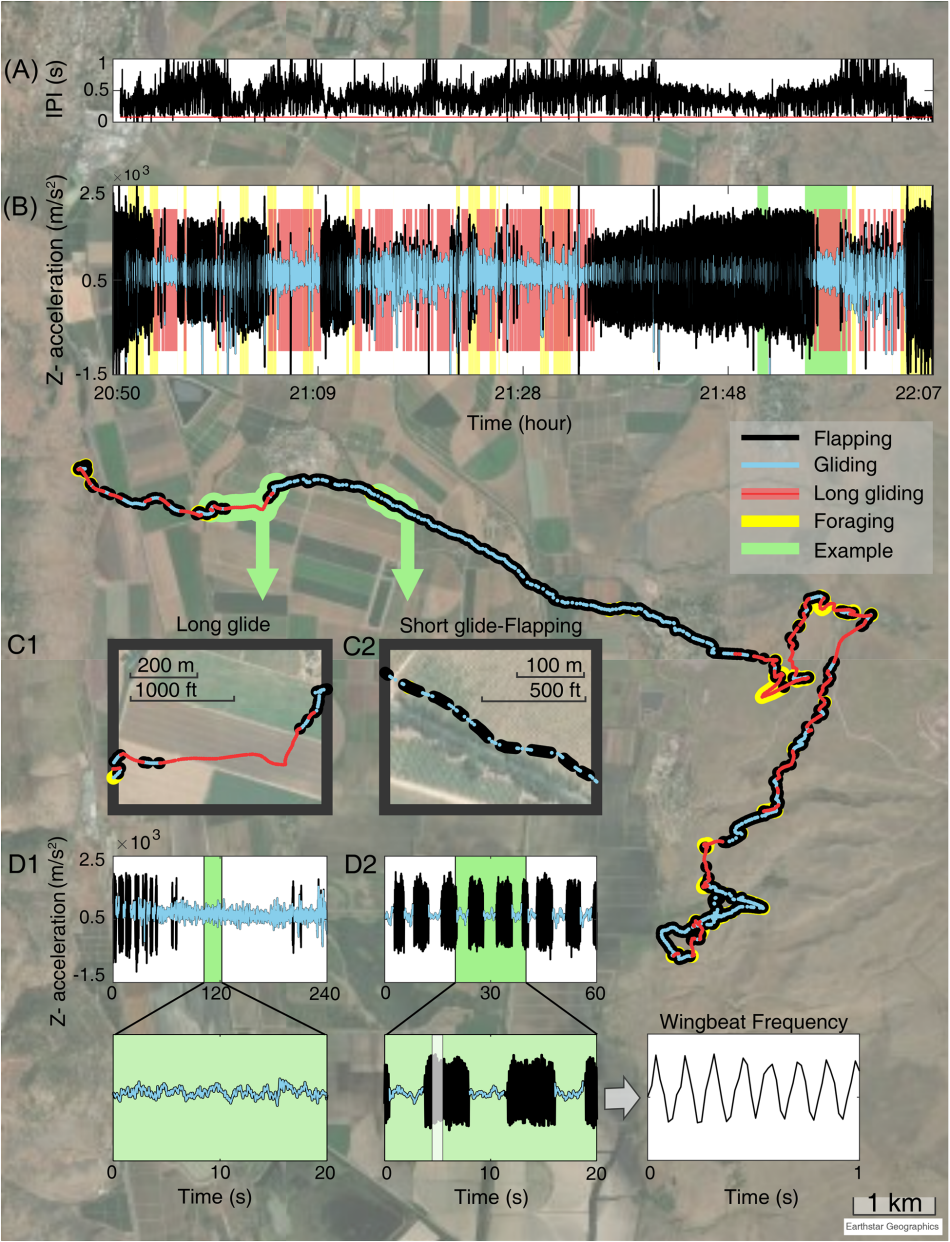
Bats constantly switch between flapping and gliding. An example of one bat's flight behavior along its flight trajectory. (A) Inter‐pulse interval (IPI) and (B) *z*‐axis acceleration for the complete flight path. The flight modes are shown over the entire path: alternating short flapping (black) and gliding modes (blue), and long gliding modes (>10 s, red rectangles). Yellow highlighting represents foraging bouts. (C1, C2) Zoomed‐in views of long gliding (C1) and flapping‐short gliding (C2) modes. (D1, D2) Zoomed‐in *z*‐axis acceleration for the long gliding (D1) and flapping‐short gliding (D2) modes with an additional zoom‐in of the wingbeat frequency.

Wind data were collected at 10‐min intervals from a ground‐based weather station in Kfar Nahum, located within the species’ foraging area. Each localization was matched to the nearest wind measurement in time. Relative wind direction (β) was calculated for each localization as the angle between the bat's movement direction and the wind direction, and applied absolute value, such that β = 0° corresponds to headwind, β = 90° to side wind, and β = 180° to tailwind. We used only data from the commuting phase for this analysis.

### 2.3 Audio Analysis

On‐board audio recordings were manually annotated using an in‐house MATLAB‐based program (Batalef), which extracted two temporal domain parameters: call duration (defined by the double‐sided 12 dB drop below the peak) and IPI for each call. Call frequency is highly correlated with call duration [35, 44, 45, 46] and was not measured here. To assign behavioral labels to calls, each call was matched to the nearest IMU peak in time via a nearest‐neighbor search. Calls inherited the flight‐mode label (flapping, gliding, long gliding) and behavioral stage (foraging vs. commuting) of the matched IMU peak and localization point, respectively.

### 2.4 Statistical Analysis

We tested the effects of three flight modes on the acoustic characteristics of the bats’ echolocation calls using generalized linear mixed‐effect models (GLMM). Two response parameters were tested: duration (normal distribution) and IPI (assuming a normal distribution after performing a log transformation). The flight mode was used as a fixed effect, and the bat's ID was set as a random effect. We compared all mode‐pairs in separate models, and performed a Benjamini−Hochberg false discovery rate correction (BH‐FDR) on the *p*‐values to account for multiple comparisons. The models were also compared to null models (with only the intercept) in a likelihood ratio test and were found to have a better fit than the null (see exceptions in Table S2). To test for differences in wind speed during the different flight modes, we fit a GLMM (gamma distribution) with the flight mode as a fixed effect and the bat's ID as a random effect.

For all experimental models, we confirmed that the residuals were approximately normally distributed by examining their distribution and the Q‐Q plots.

Model explanatory power was summarized using marginal and conditional *R* ^2^s, computed in MATLAB, following the Nakagawa and Schielzeth method [47] adapted for GLMMs.

To test for differences in the distribution of relative wind direction (β) among the various flight modes while accounting for repeated measurements from the same individuals, we used a permutation‐based approach. The test statistic quantified differences between the empirical distributions of the flight modes using the Kolmogorov–Smirnov (KS) distance. For each pair of flight modes, the KS statistic was calculated. To assess statistical significance, a permutation test was performed in which mode labels were randomly reassigned within each individual bat. This procedure preserves the within‐individual structure of the data while generating the null distribution expected in the absence of mode effects. For each permutation, the test statistic was recalculated using the permuted labels. The permutation distribution was generated from 5000 iterations, and the *p*‐value was calculated as the proportion of permutations in which the permuted statistic was equal to or greater than the observed statistic. When the overall test indicated a significant effect of mode, pairwise comparisons between modes were conducted using the same permutation procedure. Resulting *p*‐values were adjusted for multiple comparisons using the BH‐FDR correction.

To examine whether the bats initiated feeding attacks more often in a specific flight mode, we performed a GLMM with the number of attacks as the response variable (Poisson distribution with an offset for the duration of each flight mode, estimating attack rates), the flight mode was set as a fixed effect, and the bat's ID was set as a random effect. We compared mode‐pairs in separate models, and performed a BH‐FDR correction on the *p*‐values to account for multiple comparisons. All statistical analyses were performed in MATLAB R2021b.

## 3 Results

During the summer months, *R. microphyllum* foraging behavior is characterized by an initial main flight bout starting shortly after sunset, sometimes followed by one or more additional bouts later in the night, typically between midnight and 5:00 a.m. In this study, we recorded the locations of five *R. microphyllum* bats over one to three nights using ATLAS tags. We also monitored their echolocation and body acceleration during one complete bout, either at the beginning or toward the end of the night. Bats flew on average 56 ± 22 min per bout (mean ± SD, *n* = 5 bats; Table S1), covering a distance of 21 ± 6 km. We defined two behavioral stages of flight: (1) commuting, characterized as straight flight, and (2) foraging, characterized by tortuous flight (not including feeding events, which were analyzed separately; see Methods). The bats spent, on average, 84 ± 13% of the flight commuting and 16 ± 13% of the flight foraging.

All bats used intermittent flap‐gliding flights, alternating between flapping and short gliding modes during both commuting and foraging (Figure 1). Flapping and short gliding bout durations were 3 ± 1.4 s and 2.2 ± 0.3 s, respectively, during commuting and 1.5 ± 0.8 s and 1 ± 0.6 s, respectively, during foraging. Four of the five bats exhibited an additional flight mode, using long gliding bouts, lasting longer than 10 s, during both commuting (31.5 ± 14.5 s long, *n* = 4 bats) and foraging (28 ± 6 s long, *n* = 3 bats). The longest gliding bout lasted 232 s. Long glides accounted for up to 24 ± 22 (*n* = 4 bats) and 52 ± 14 (*n* = 3 bats) percent of the total commuting and foraging flight duration, respectively (Figure 2A). We note that in total, the bats spent more time in long glides during commuting, even though the proportion of long glides during foraging was higher, because the commuting accounted for 84% of the total time of their flight. During long gliding, the wind speed (measured at ground level) was significantly lower than during flapping and short gliding (GLMM, *p* < 0.001 for both commuting and foraging; Table S2); however, the difference was small (< 0.3 m/s), with an overall low wind speed during all recording days (1.9 ± 1.3 m/s). Despite the low wind speeds, our results suggest that the bats used different flight modes according to the wind direction. We calculated the angle between the bat's flight direction and the wind direction (at ground level) and found that the bats used long gliding more often when experiencing side‐tail wind (110−160 degrees) compared to both short gliding and flapping flights, which were performed nearly uniformly across wind directions (permutation KS test *p* < 0.001 for short vs. long gliding and flapping vs. long gliding, and *p* = 0.04 for short gliding vs. flapping; Figure S7).

**FIGURE 2:**
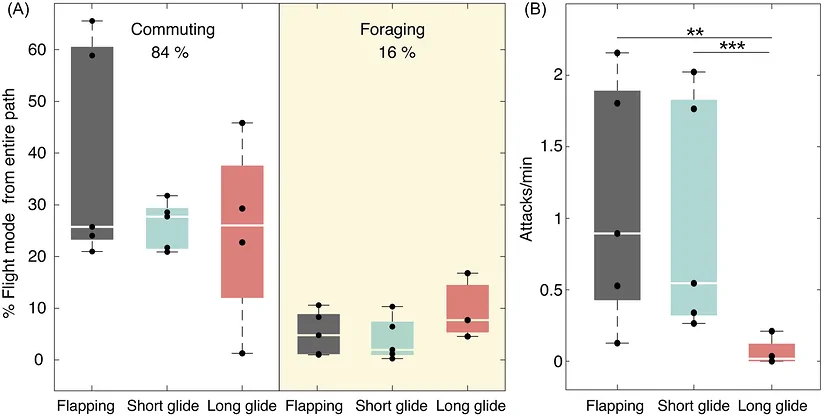
Flight mode propensity and feeding attacks. (A) The percentage of time the bats spent in each flight mode, during commuting (left) and foraging (right). Commuting accounted for 84% of the entire flight path and foraging 16%. Note that flight mode percentages are relative to the entire flight path (i.e., LG: 9% of total flight time is 52% of foraging flight time). (B) Number of attacks per minute initiated during each flight mode (defined as the number of initiated attacks divided by the total time the bat spent in the specific flight mode), for commuting and foraging combined. Asterisks in panel B represent a significant difference between the groups (*p* ≤ 0.0005, *** *p* ≤ 0.00005). Boxes represent the lower and upper quartiles, lines represent the median, and dots represent individual data points (bats).

Throughout the flight path, the bats initiated attack sequences, characterized by shortening the intervals between calls (see Methods for definition), approaching toward the potential capture of an insect. These feeding events occurred nearly exclusively during the flapping and short gliding modes (97 ± 2.8% of all attacks, Table S3) rather than during long glides (GLMM, *p* < 0.001 after BH‐FDR correction for multiple comparisons; Table 1 and Figure 2B) across the entire flight path (commuting and foraging combined).

**TABLE 1:** Statistical results.

Flight phase	Response	Fixed effect	*p*‐value	Estimate	SE	*R* ^2^m/*R* ^2^c	Sample size (calls)	AIC
	**Acoustic feature ∼ 1 + flight mode + (1|Bat)**							
**Commuting**	**IPI** (log transformed)	SG—F	**< 0.001**	0.04	0.003	0.005/0.2	27,164	−6192.7
		SG—LG	**< 0.001**	−0.04	0.003	0.007/0.2	17,570	−5114.5
		F—LG	**< 0.001**	−0.08	0.003	0.023/0.2	22,564	−8205
	**Duration**	SG—F	**< 0.001**	0.0003	2.3e−05	0.004/0.1	27,394	−266,520
		SG—LG	**< 0.001**	−0.0004	2.7e‐05	0.012/0.1	17,720	−177,210
		F—LG	**< 0.001**	−0.0006	2.8e‐05	0.023/0.2	22,752	−224,540
**Foraging**	**IPI** (log transformed)	SG—F	**< 0.001**	0.1	0.006	0.06/0.2	4952	−2306
		SG—LG	**< 0.001**	−0.06	0.006	0.021/0.2	5025	−2167
		F—LG	**< 0.001**	−0.17	0.005	0.14/0.3	5979	−3053
	**Duration**	SG—F	**< 0.001**	0.0009	6e‐05	0.035/0.3	4984	−47,995
		SG—LG	**< 0.001**	−0.0007	5.1e‐05	0.025/0.3	5067	−49,957
		F—LG	**< 0.001**	−0.0015	5.6e‐05	0.1/0.3	6017	−57,980
	**Feeding attacks ∼ 1 + flight mode + (1|Bat)**							
	**Feeding attacks** (Poisson distribution)	LG—F SG—F	**< 0.001** **0.2**	−2.9 −0.2	0.42 0.14		14	39
		SG—LG	**< 0.001**	2.7	0.42		9	33

*Note*: Statistical models were fit for flight mode‐pairs between flapping (F), short gliding (SG), and long gliding (LG). p‐values are shown after BH‐FDR correction for multiple comparisons. Unless stated otherwise, models were fitted with a normal distribution. For *p*‐values < 0.05, marginal (*R* ^2^m) and conditional (*R* ^2^c) *R* ^2^ are shown for the variance explained by the fixed effect (flight mode) and the fixed and random effects (flight mode + bat ID), respectively. For feeding attacks—Sample size refers to the number of observations (bats × phase).

**TABLE 2:** Means and standard deviation of acoustic parameters in the different flight modes.

Flight phase	Flight mode	Duration (s) (mean ± SD)	IPI (s) (mean ± SD)
**Commuting**	**Flapping**	0.014 ± 0.0008 (*n* = 5)	0.35 ± 0.09 (*n* = 5)
	**Short glide**	0.014 ± 0.0006 (*n* = 5)	0.4 ± 0.1 (*n* = 5)
	**Long glide**	0.014 ± 0.001 (*n* = 4)	0.35 ± 0.09 (*n* = 4)
**Foraging**	**Flapping**	0.012 ± 0.002 (*n* = 5)	0.25 ± 0.06 (*n* = 5)
	**Short glide**	0.013 ± 0.001 (*n* = 5)	0.33 ± 0.08 (*n* = 5)
	**Long glide**	0.014 ± 0.0008 (*n* = 3)	0.35 ± 0.08 (*n* = 3)

*Note*: Sample size (*n*) refers to the number of bats.

Next, we compared the bat's echolocation signals in the three flight modes. We extracted two acoustic parameters from the on‐board recordings: signal duration and IPI (Table 2). We used generalized linear mixed models (GLMMs with BH‐FDR correction for multiple comparisons, see Methods) to test for acoustic differences between the flight modes. Signal duration and IPI were significantly higher during long gliding and lowest during flapping in both commuting and foraging (GLMMs, *p* < 0.001 for all three mode‐pairs; see Table 1 and Figure 3). The most substantial adjustment to these parameters occurred during long glides compared to flapping, during foraging, where there was an ∼11% increase in call duration and a ∼15% increase in IPI, relative to the parameter's mean (marginal *R* ^2^ = 0.1 and *R* ^2^ = 0.14, respectively).

**FIGURE 3:**
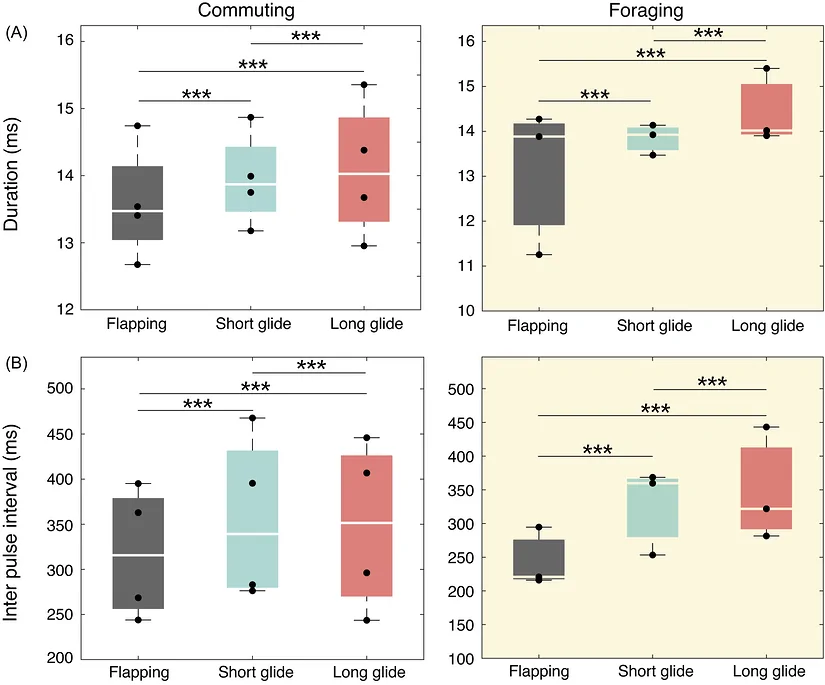
Acoustic parameters. Boxplots of (A) signal duration and (B) IPI in the different flight modes during commuting (left, *n* = 4 bats) and foraging (right, *n* = 3 bats). Data are shown only for the bats that exhibited all three flight modes. Boxes represent the lower and upper quartiles, lines represent the median, and dots represent individual data points (bats). An asterisk represents a significant difference between the groups (*** *p* ≤ 0.00005).

## 4 Discussion

Intermittent flap‐gliding flights have been anecdotally observed in bats in the field but have only been recorded in a few species [16, 17, 18]. Here, we used on‐board tracking to show that under natural conditions, greater mouse‐tailed bats use intermittent flap‐gliding flights in addition to long gliding bouts throughout their flight path. Intermittent flap‐gliding birds spend different proportions of time gliding, and in many cases, conserve energy compared to continuous flapping flight [11, 29, 30, 31, 32]. *R. microphyllum* bats glided ∼ 50% of their overall flight time, during both commuting and foraging. Comparative aerodynamic models predict different energetic consequences of flap‐gliding between birds and bats. Modeling work combining empirical lift‐to‐drag ratios for flapping and gliding flight indicates that, whereas the common swift (*Apus apus*) can reduce transport costs by alternating between these modes, the lesser long‐nosed bat (*Leptonycteris yerbabuenae*) may be unable to achieve similar energetic savings [11]. This discrepancy has been attributed to differences in wing morphology and aerodynamic efficiency during gliding. A more recent aerodynamic model based on bat flight measurements of three species that regularly employ undulating flight (*R. microphyllum, Nyctalus noctula*, and *Taphozous theobaldi*) posits that there is a similar advantage of intermittent flap‐gliding flight in bats to that of birds [12]. *R. microphyllum*’s frequent use of gliding suggests that these bats may be able to exploit aerodynamic conditions to reduce transport costs or enhance flight efficiency. Their high aspect ratio and fast flight speeds support their adaptability to flap‐gliding flight, as these parameters contribute to greater energy savings in gliding birds [32]. It would be interesting for future studies to confirm whether they experience an energetic advantage similar to that of gliding birds.

Bats are known to couple their echolocation emission to the wingbeat in order to reduce echolocation production costs during flapping flight [27, 28]. While gliding, the echolocation production is uncoupled from the wingbeat during exhalation, which should increase the cost of call production, especially for loud calls [48] as emitted by this species. Partial compensation for the energetic costs could be achieved by reducing the calling rate. A review of the acoustic characteristics of the bats’ echolocation in the different flight modes revealed that the bats reduced emission rate during long gliding bouts. This decrease in call emission rate could also result from a shift in behavior from prey search and attack (which benefits from calling more) to rest or changes in exhalation during gliding [49]. We also found that the bats initiated prey‐attack sequences almost exclusively during flapping and short gliding, likely investing less in prey search during long glides, as also indicated by the longer intervals between calls. On very few occasions (< 5%), the bats initiated an attack immediately after a long glide (within 1 s), suggesting that they are still attentive to prey echoes during these periods even if not fully invested in search.

Exploiting the wind is another way to save energy in flight. Soaring migrant birds are able to lower their airspeed under tailwind conditions, thus covering more ground while conserving energy [50]. Other migrating birds were shown to adjust their daily flight distance depending on whether the wind conditions were favorable or not (tailwind or headwind) [51]. Bats are also able to time their migration and adjust their flight speed and altitude to accommodate different wind conditions [17,52, 53,54]. Fruit bats reportedly adjust their airspeed in accordance with tailwind to allow a steady flight speed throughout their flight path as well as compensate for crosswind effects [52]. In this study, we found a small effect of wind speed and direction on flight behavior, consistent with expectations: long gliding was performed preferably in low side and tailwinds, while flapping in headwinds. Wind speed and direction were only measured at ground level, and wind speed was relatively low on all recording days, so the effect of the wind on the bat's momentary flight mode would require further investigation.

Key questions regarding gliding flight in bats remain unresolved. The prevalence of gliding across bat taxa, the flight conditions under which it is employed, and its aerodynamic and energetic consequences are still incompletely characterized. The persistence of gliding or flap‐gliding behaviors in extant bats may represent not merely a vestigial trait, but a functional component of their flight strategy. Addressing these questions is essential for a more complete understanding of bat flight performance.

## 5 Conclusions

The greater mouse‐tailed bat uses short and long gliding bouts as a substantial part of their flight behavior. Gliding accounted for ∼ 50% of the total flight duration and occurred during both commuting and foraging flights. Our results reveal that long gliding bouts may represent a different mode of behavior during which the bats may conserve energy by taking advantage of the weather conditions and calling less often.

## Author Contributions

A.G. and Y.Y. conceived and designed the research. A.G. and Y.O. collected the data. A.G. and M.T. analyzed the data. Y.O., S.T., and R.N. developed and implemented the ATLAS system. M.T., A.G., and Y.Y. wrote the manuscript. All authors read and approved the final manuscript.

## Funding

This study was partially supported by a grant from the Israel Science Foundation [331/22, Y.Y.], a grant from the Simons Foundation International [SFI‐LS‐SEFF‐00017081, A.G.], the Minerva Center for Movement Ecology [R.N.], and the Israel Science Foundation [965/15, S.T.].

## Conflicts of Interest

The authors declare no conflicts of interest.

## Supporting information




**Supplementary Material**: nyas70416‐sup‐0001‐SuppMat.docx

## Data Availability

Data reported in this paper have been deposited at Mendeley Data (https://doi.org/10.17632/xkgbhb5b6s.1 and will be available upon publication.
