# Supplementary material for: Greater Mouse‐Tailed Bats Glide During Approximately 50% of Their Flight
Source: Ann N Y Acad Sci. 2026 Oct 13;1564(1):e70416. doi: 10.1111/nyas.70416 (PMC13660705; doi:10.1111/nyas.70416)
Supplement: Supplementary file 1 [file NYAS-1564-0-s001.docx]

**Supplementary materials**

**Table S1: Audio and acceleration recording dates and times.**

|  | **Recording date** | **Start time** | **End time** | **Flight time (min)** |
| --- | --- | --- | --- | --- |
| **Bat 3** | 18.06.2018 | 02:14:04 | 02:49:30 | 35.4 |
| **Bat 8** | 18.06.2018 | 00:04:01 | 00:34:02 | 30 |
| **Bat 11** | 17.06.2018 | 20:50:00 | 22:07:38 | 77.6 |
| **Bat 24** | 07.08.2018 | 19:59:42 | 21:16:25 | 76.7 |
| **Bat 26** | 07.08.2018 | 19:58:31 | 20:58:43 | 60.2 |

**Table S2: Additional statistical models.** Short glide (SG), long glide (LG), flapping (F). Null models were compared to the full models in likelihood ratio tests (LRT).

|  | **Response** | **Fixed effect**  **(data set)** | **P-value** | **Estimate** | **LRT P-value** | **LRT Chi-square** | **Sample size** | **AIC** |
| --- | --- | --- | --- | --- | --- | --- | --- | --- |
| **commute** | **Wind speed**  (Gamma distribution) | SG - F  F - LG  SG - LG | 0.3  **< 0.001**  **< 0.001** | -0.009  0.12  0.11 |  |  | 3499  1903  1816 | 1178  676  679 |
|  | **Null models** | | | | | | | |
|  | **IPI**  (Log transformed) | Intercept (SG-F) | **< 0.001** | -0.5 | **< 0.001** | 176 | 27164 | -6018 |
|  |  | Intercept (SG-LG) | **< 0.001** | -0.5 | **< 0.001** | 130.7 | 17570 | -4986 |
|  |  | Intercept (F-LG) | **< 0.001** | -0.5 | **< 0.001** | 529.3 | 22564 | -7677 |
|  | **Duration** | Intercept (SG-F) | **< 0.001** | 0.014 | **< 0.001** | 132.1 | 27394 | -266390 |
|  |  | Intercept (SG-LG) | **< 0.001** | 0.014 | **< 0.001** | 222.6 | 17720 | -176990 |
|  |  | Intercept (F-LG) | **< 0.001** | 0.014 | **< 0.001** | 497.4 | 22752 | -224050 |
|  | **Wind speed**  (Gamma distribution) | Intercept (SG-F)  Intercept (SG-LG)  Intercept (F-LG) | **0.16**  **0.15**  **0.16** | -0.2  -0.2  -0.2 | **0.45**  **< 0.001**  **< 0.001** | 0.6  17.8  19.6 | 3499  1816  1903 | 1176  694.5  694 |
| **Foraging** | **Wind speed**  (Gamma distribution) | SG - F  F - LG  SG - LG | **0.03**  **< 0.001**  **< 0.001** | -0.04  0.26  0.22 |  |  | 1124  617  593 | 318.5  255.5  226 |
|  | **Null models** | | | | | | | |
|  | **IPI**  (Log transformed) | Intercept (SG-F) | **< 0.001** | -0.6 | **< 0.001** | 316.7 | 4952 | -1992 |
|  |  | Intercept (SG-LG) | **< 0.001** | -0.53 | **< 0.001** | 123.8 | 5025 | -2045 |
|  |  | Intercept (F-LG) | **< 0.001** | -0.6 | **< 0.001** | 875.5 | 5979 | -2180 |
|  | **Duration** | Intercept (SG-F) | **< 0.001** | 0.013 | **< 0.001** | 214.7 | 4984 | -47782 |
|  |  | Intercept (SG-LG) | **< 0.001** | 0.013 | **< 0.001** | 176.7 | 5067 | -49783 |
|  |  | Intercept (F-LG) | **< 0.001** | 0.013 | **< 0.001** | 663 | 6017 | -57319 |
|  | **Wind speed**  (Gamma distribution) | Intercept (SG-F)  Intercept (SG-LG)  Intercept (F-LG) | **0.35**  **0.3**  **0.4** | -0.2  -0.2  -0.2 | **0.01**  **0.003**  **< 0.001** | 6.2  8.6  10.9 | 1124  593  617 | 323  233  264 |
|  | **Feeding attacks**  (Poisson distribution) | Intercept (LG-F; SG-F) | **0.004** | 1.1 | **< 0.001** | 160 | 82.3 | 117 |
|  |  | Intercept (SG-LG) | **0.03** | 0.8 | **< 0.001** | 117 | 68.2 | 99 |

**Table S3: Mean and standard deviation of attacks per minute initiated during the different flight modes.** Attacks/minutes are defined as the number of initiated attacks divided by the total time the bat spent in the specific flight mode.

|  | **Flapping** | **short glide** | **Long glide** |
| --- | --- | --- | --- |
| **Bat 3** | 0.13 | 0.26 | 0 |
| **Bat 8** | 0.53 | 0.54 | NaN |
| **Bat 11** | 0.9 | 0.34 | 0.03 |
| **Bat 24** | 1.8 | 1.76 | 0 |
| **Bat 26** | 2.15 | 2 | 0.2 |
| **Mean ± SD** | **1.1 ± 0.8** | **1 ± 0.8** | **0.06 ± 0.1** |

**Figure S1: Movement analysis****.** Example bat trajectory illustrating the data cleaning and smoothing pipeline. The bats' localization was sampled every 1–2 Hz; however, not all samples yielded successful localizations, and the raw data (yellow) included both low- and high-quality localizations. Before analyzing movement, low-quality localizations were filtered based on a confidence score derived from the number of stations and localization error; only high-confidence points were retained, and additional spatial and speed outliers were removed (black arrows), yielding the cleaned track (black). This filtering resulted in an effective median sampling interval of 4 s across the five bats. The cleaned track was subsequently smoothed with a LOESS filter (9-point window) and linearly interpolated to yield one position every 4 s, producing the final smoothed and interpolated trajectory (red). The inset panel shows a zoomed view of a representative segment where raw localization noise was most pronounced. Basemap imagery: Earthstar Geographics.

**Figure S2: Segmentation of bats’ movement between commuting and foraging** **using a straightness index (SI).** **(A)** SI distribution for bat 3, exhibiting both commuting and foraging behavior during its track, revealing a long-tail (nearly bimodal) pattern. This bimodality is consistent with previous work on this species (Cvikel et al., 2015), in which bats exhibit both commuting and foraging behavior, supporting the use of SI to segment flight trajectories into commuting (SI > 0.5) and foraging (SI < 0.5) behavior. **(B)** Flight trajectory of bat 3, with commuting (white) and foraging (red) segments classified using this threshold. The inset panel shows a zoomed-in view of movement within one foraging patch. Basemap imagery: Earthstar Geographics.

**Figure S3: Z-axis acceleration and echolocation activity during commuting flight.** **(A)** Z-axis body acceleration over a representative 30-second window of commuting flight, showing alternating bouts of flapping (black) and gliding (blue). Red vertical lines mark the timing of the focal bat's echolocation calls. The green-shaded region indicates the 4-second window expanded in panel B. **(B)** Zoomed-in view of the shaded region in panel A, showing acceleration during flapping and gliding in greater detail; red lines again mark the focal bat's echolocation calls (example indicated by the arrow). **(C)** Spectrogram of the audio recording corresponding to the same 4-second window shown in panel B. The green box indicates the 1-second segment expanded in panel D. **(D)** Spectrogram of the audio recording during the 1-second segment outlined in panel C. Red and blue arrows indicate echolocation calls produced by the focal bat and by conspecifics, respectively; yellow arrows indicate flapping-associated noise.

**Figure S4: Z-axis acceleration and echolocation activity during foraging flight.** **(A)** Z-axis body acceleration over a representative 30-second window of foraging flight, showing alternating bouts of flapping (black) and gliding (blue). X-axis and y-axis body acceleration is presented in dark blue and dark green, respectively, and flight speed is presented in magenta (right y-axis). Red vertical lines mark the timing of the focal bat's echolocation calls. The light green-shaded region indicates the 6-second window expanded in panel B. **(B)** Zoomed-in view of the shaded region in panel A, showing z-axis acceleration during flapping and gliding in greater detail; red lines again mark the focal bat's echolocation calls (example indicated by the arrow). **(C)** Spectrogram of the audio recording corresponding to the same 6-second window shown in panel B. The green box indicates the 1-second segment, corresponding to a feeding attack, expanded in panel D. **(D)** Spectrogram of the audio recording during the 1-second feeding attack outlined in panel C. Red and blue arrows indicate echolocation calls produced by the focal bat and by conspecifics, respectively; yellow arrows indicate flapping-associated noise.

**Figure S5**: **Classification of flight modes. (A)** Classification of zACC peaks into flapping and gliding. The distribution of zACC peak prominence for each bat during the entire flight trajectory, with flight mode indicated by color (black for flapping and green for gliding). Peak prominences were grouped into two categories using k-means clustering based on each bat's values. **(B)** Distribution of gliding bout durations across all five bats. The green line shows the mean proportion of gliding bouts per duration bin; the shaded green region indicates ±1 SD. The distribution declines steeply between 0 and 7 s and flattens into a low, stable tail at ~10 s, with no additional structure evident at longer durations. The dashed red line marks the 10-second threshold used to classify short versus long gliding bouts. Although the trend remains low and flat throughout this range, the x-axis is truncated at 30 s for clarity; the maximum observed gliding duration in the full dataset was 232 seconds.

**Figure S6: Classification of feeding attack sequences. (A)** Spectrograms of call sequences recorded on-board. Upper panel shows a zoom-in on the initiation of a feeding approach into attack, with decreasing IPI. **(B)** Distribution of IPIs in the recorded data. Feeding events were classified by an IPI smaller than 80 ms.

**Figure S7: Effect of wind direction on the flight mode.** Polar plot of the distribution of the angle between the bat’s flight direction and the wind direction (0, 90, and 180 degrees represent head, side, and tail wind, respectively; Wind measured at ground level) in the three flight modes (mean, n = 4 bats) during commute flight.
